# A Low-Profile Circularly Polarized Millimeter-Wave Broadband Antenna Analyzed with a Link Budget for IoT Applications in an Indoor Scenario

**DOI:** 10.3390/s24051569

**Published:** 2024-02-29

**Authors:** Parveez Shariff Bhadravathi Ghouse, Pallavi R. Mane, Tanweer Ali, Goutham Simha Golapuram Dattathreya, Sudheesh Puthenveettil Gopi, Sameena Pathan, Jaume Anguera

**Affiliations:** 1 Department of Electronics and Communication Engineering, Manipal Institute of Technology, Manipal Academy of Higher Education, Manipal 576104, India; parveez.bg@learner.manipal.edu (P.S.B.G.); palvi.mane@manipal.edu (P.R.M.); goutham.simha@manipal.edu (G.S.G.D.); sudheesh.pg@manipal.edu (S.P.G.); 2 Department of Information and Communication Technology, Manipal Institute of Technology, Manipal Academy of Higher Education, Manipal 576104, India; sameena.bp@manipal.edu; 3 Ignion, 08174 Barcelona, Spain; jaume.anguera@salle.url.edu; 4 Research Group on Smart Society, La Salle Engineering, Universitat Romon Llull, 08022 Barcelona, Spain

**Keywords:** CMA, circular polarization, IoT, link margin, millimeter-wave antenna

## Abstract

Broadband antennas with a low-profile generating circular polarization are always in demand for handheld/ portable devices as CP antennas counter multipath and misalignment issues. Therefore, a compact millimeter-wave antenna is proposed in this article. The proposed antenna structure comprises two circular rings and a circular patch at the center. This structure is further embedded with four equilateral triangles at a 90° orientation. The current entering the radiator is divided into left and right circular directions. The equilateral triangles provide the return path for current at the differential phase of ±90°, generating circular polarization. Structural development and analysis were initially performed through the characteristic mode theory. It showed that Modes 1 to 4 generated good impedance matching from 20 to 30 GHz and Modes 1 to 5, from 30 to 40 GHz. It also demonstrated the summation of orthogonal modes leading to circular polarization. The antenna-measured reflection coefficient |S11| > 10 dB was 19 GHz (23–42 GHz), and the axial ratio at −3 dB was 4.2 GHz (36–40.2 GHz). The antenna gain ranged from 4 to 6.2 dBi. The proposed antenna was tested for link margin estimation for IoT indoor conditions with line-of-sight (LOS) and non-line-of-sight (NLOS) conditions. The communication reliability with co- and cross-polarization was also studied under these conditions, and the results proved to be satisfactory.

## 1. Introduction

Millimeter-wave (mmWave) spectrum potential is currently being exploited in the fields of Internet of things (IoT) [1], vehicular communication [2], radio astronomy [3], mobile communications [4], energy harvesting [5], satellite communication [6], millimeter-wave imaging [7], etc. It endeavors to deliver higher bandwidth with low latency and a significant data rate. However, the mmWave poses several challenges to the propagation of signals, causing higher path loss due to atmospheric attenuations. Nevertheless, for short-range communication, the mmWave performs well. The antenna characterization of a mmWave is crucial for efficient and reliable communication. With the increased wavelength at an mmWave, the form factor of the antenna profile becomes highly compact, which makes embedding the antenna on handheld devices and chips feasible [8]. Broadband antennas are the preferred choice to cover the huge spectrum of the mmWave. The fading of the mmWave spectrum is significant, where CP antennas play a vital role in mitigating multipath signals [9]. Also, CP antennas are preferred over linearly polarized (LP) antennas as they provide the solutions to antenna misalignment, polarization change due to multipath, and attenuation to rain and snow. Therefore, broadband and CP can be realized with structures such as a magneto–electric dipole [6], dielectric resonator (DRA) [1], substrate integrated-waveguide (SIW) loop antennas [10], and planar dipole antennas fed by an SIW [11].

However, the antenna profile of these structures is large for IoT handheld or portable devices. Therefore, the pursuit of simple, planar, cost-efficient CP antennas is always in demand. In [12], an E-shaped patch structure whose bandwidth was increased by feeding the signal through an L-shaped probe is proposed. Similarly, in [13], an L-shaped probe was used to feed a signal to an arrangement of six square parasitic elements which were arranged in a 2 × 3 fashion. The arrangement of parasitic elements with a suitable distance achieved a broad bandwidth of 7.5 GHz (21–28.5 GHz). Both Refs. [12,13] were able to achieve broadband with LP but not CP. A metamaterial-based metasurface also aids in attaining wider bandwidth, like in [14]. Here, a circular patch was the main radiating element fed by a coaxial feed. The radiator was optimized by truncating with two semi-circles that were further surrounded by square ring resonators which behaved as a metasurface acting as secondary resonators that enhance the bandwidth. Due to the truncation of the circular patch and surrounding metasurfaces, CP was achieved. In [15], a planar structure with two hexagonal rings and a partial ground is proposed. The electrical length of the outer and inner rings was (3/4)λ and λ at 28 and 38 GHz, respectively, resulting in dual resonance with broad bandwidth. A monopole antenna with a grounded coplanar waveguide (CPW) can also achieve wider bandwidth. In [16], a monopole antenna with a ground-CPW is proposed. Here, the CPW had a couple of slots on either side and further along the monopole’s two pairs of parasitic stubs. The combination of these resulted in 2 GHz of bandwidth from 27 to 29 GHz. In [17], a conventional approach was used to achieve CP. In this case, the edges of rectangular patch were trimmed, and a signal was fed from the feed line, which was at the bottom layer through via. The structure achieved a wide bandwidth with left-hand CP. 

The above literature reveals a wide variety of structures lead to broadband with CP; however, most of the designs have large antenna profiles and are multi-layered which motivated us to design a compact broadband CP antenna. Therefore, this paper presents a miniaturized broadband antenna with right-hand and left-hand CP characteristics. The radiating element combines two circular rings, a circular patch, and two sets of embedded triangular stubs. The ground plane is truncated with I-shaped stubs at either vertical edge for impedance matching. The structural development of the antenna proceeded with the characteristic mode theory (CMA). This method is meticulously used for complex structures to comprehend the surface current on the conducting surface of the antenna and its resultant modes and radiation pattern. Also, in this article, we performed a link margin estimation of the proposed antenna for IoT applications in an indoor scenario.

## 2. Characteristic Theory-Based Antenna Methodology

The characteristic mode analysis (CMA) has been applied to arbitrary complex antenna structures where the mathematical proofing of such structures becomes difficult. It determines the naturally resonant modes and position of excitation on the antenna structure. Initially, Garbacz [18,19] proposed this method, which was later improved by Harrington and Mautz [20], who applied integral equations (IEs) to derive surface currents. An IE is solved for independent surface current modes that result closely to the impedance matrix obtained using the method of moments (MoM) [21]. For our case, analysis was performed by considering the impinging incident wave on a perfectly conducting surface and lossless substrate material. An eigenvalue ($\lambda_{n})$ with a modal excitation coefficient ($V_{n}^{i})\;$ relates the electric field ($E^{i})$ and surface current ($J_{n})$ on the structure. This coefficient provides adequate information on the feed position, magnitude, and phase effects of individual modes resulting in total surface current [22]. Therefore, the total surface current on the conductor surface due to the incident wave was computed using Equation (1):

(1) $$J=\underset{n}{\sum }a_{n}J_{n}=\underset{n}{\sum }\frac{V_{n}^{i}J_{n}}{1+j\lambda_{n}}=\underset{n}{\sum }\frac{\langle J_{n},\;E^{i}\rangle J_{n}}{1+j\lambda_{n}}\;\;\;$$

where $a_{n}$ is the excitation coefficient of the *n*-th naturally resonant mode. When the eigenvalue ($\lambda_{n}$) approaches zero, this indicates that the resulting individual surface current/mode is significant, which is estimated using Equation (2):

(2) $$MS_{n}=\left|\frac{1}{1+j\lambda_{n}}\right|$$

However, structural properties and their behavior can be further studied through relative phase differences between these modes with the aid of the characteristic angle (CA). The incident electric field ($E^{i})$ on the conductor induces the surface current ($J_{n}),$ which is relayed by Equation (3) [22]:

(3) $$E^{i}={∭}_{V_{n}^{i}}J_{n}\cdot \bar{G}dv^{i}$$

where $\bar{G}$ is the dyadic Green’s function for the current source $J_{n}$. Based on the material property and construction of the structure, phase lag may occur between the oscillating electric field and induced surface current due to the structure’s capacitance or inductance effect. The resulting phase difference is calculated from the eigenvalue ($\lambda_{n})$ using Equation (4):

(4) $$\varphi =180{}^{\circ}-tan^{-1}\lambda_{n}$$

Here, the phase difference between 90° and 180° indicates that the structure has an inductance effect that generates opposing electromotive force (EMF) in response to the incident electric field, causing phase delay in the oscillating mode surface current ($J_{n}$). This phase lag consequently stores the energy in magnetic form, causing no radiation. On the other hand, the phase difference of 180° to 270° indicates the structure stores electric charges in an incident electric field, leading to a capacitance effect. When φ=180°, the structure has no capacitance or inductance effect for that respective mode, and thus, the mode is naturally resonant and radiating completely. In [23], a series of H-shaped and metallic strip metasurface characteristics were studied through CMA. The distance and dimension of the H-shaped structure was optimized by analyzing its resultant eigenvalue $\left(\lambda_{n}\right)$. In another article, ref. [24], the CP behavior of a slotted U-shaped antenna was analyzed by observing the direction of surface current using CMA.

**Figure 1:**
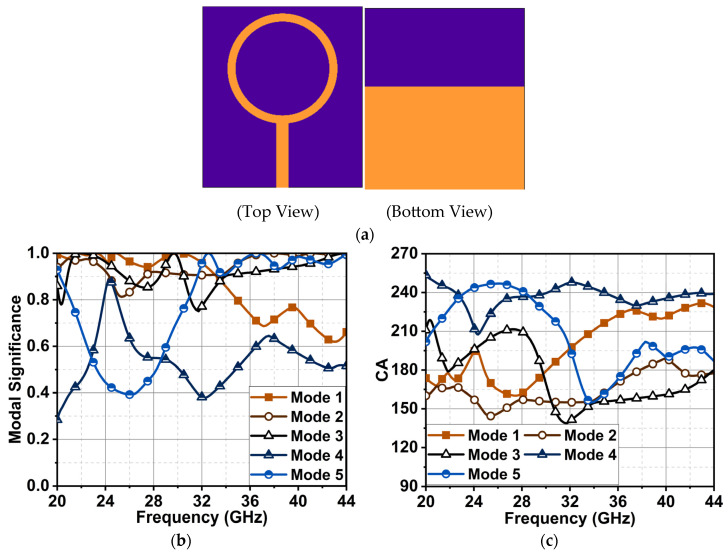
(**a**) The antenna structure of a simple ring (Ant1) and its results: (**b**) modal significance difference and (**c**) characteristic angle difference.

In our case, the final antenna structure evolved with modification of the circular ring shown in Figure 1a. It was designed on a Rogers 5880 substrate with a thickness of 0.254 mm.

The CMA of the circular ring of Figure 1a sorted it at 28 GHz with five modes. CMA indicated that Mode 1 was significant from 20 to 34 GHz, considering a modal significance at 0.8. However, Modes 2 and 3 were significant from 20 to 44 GHz. Mode 4 had a narrow bandwidth from 24 to 25 GHz, and Mode 5 attained significance from 29 to 44 GHz, as depicted in Figure 1b. The phase difference between these modes could be analyzed with a characteristic angle in Figure 1c. It is worth noting that the structure has an inherent inductance and capacitance nature, which is reflected through its various modes when an electric field is incident. Modes 1 and 2 generated an opposing EMF for an incident field ($E^{i})$, leading to inductance from 20 to 30 and 20 to 37 GHz. A similar effect was observed for Mode 3 from 30 to 44 GHz. However, Mode 1 changed its character from inductance to capacitance from 30 to 44 GHz, and Mode 4 was largely capacitance. The zero-phase lag that was a CA of 180° was observed at 22 GHz, where Modes 1, 2, and 3 had almost zero phase differences. Consequently, this may have resulted in a good impedance match. Also, at 30 GHz, Modes 1 and 3 had zero-phase lag (i.e., at CA = 180°); however, Modes 2, 4, and 5, which had a huge phase lag, created an impedance mismatch, impacting the antenna performance. 

**Figure 2:**
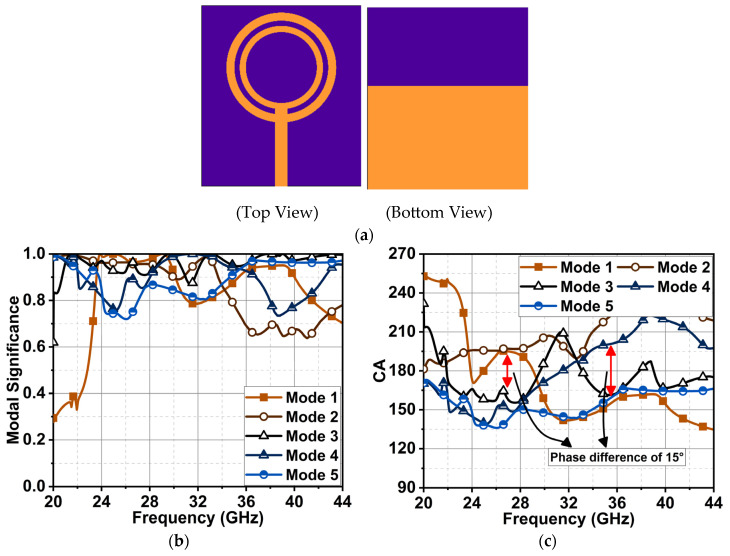
(**a**) The antenna structure of two concentric rings (Ant2) and their results: (**b**) MS difference and (**c**) CA difference.

Further, these modes’ behavior was improved by adding another circular ring inside the outer ring to the antenna structure in Figure 2a. In this case, all the modes appeared significant from 24 to 34 GHz, and Modes 1, 3, 4, and 5 were prominent from 34 to 42 GHz, as shown in Figure 2b. However, a phase lag of 15° (i.e., 180° ± 15°) was seen from 23 to 28.5 GHz for Modes 1 and 2 with a capacitance effect and Modes 3 to 5 with an inductance effect, as depicted in Figure 2c. Moreover, these modes exhibited non-linear behavior for changes in frequency. 

**Figure 3:**
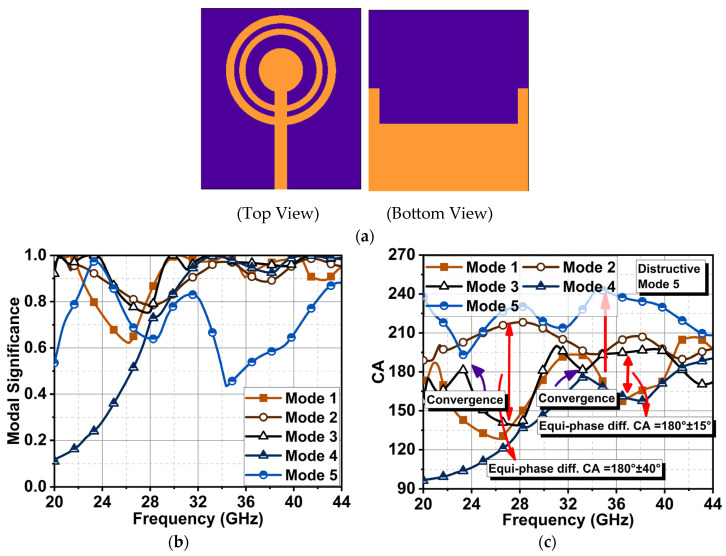
(**a**) Concentric ring antenna added with another circular patch at the center of the radiator (Ant3). The corresponding (**b**) MS and (**c**) CA difference results.

The non-linearities in the modes were corrected by adding a circular patch inside the inner ring, which terminated the feed line. Also, the conductive area of the ground plane was reduced, excluding the vertical edge portion, as illustrated in Figure 3a. The resultant MS in Figure 3b revealed that Modes 1, 2, 3, and 5 were dominant from 20 to 23 GHz; beyond this frequency, Modes 2 and 3 remained significant until 27 GHz. From 27 GHz onwards, a linear response of Modes 1 to 4 could be observed until 44 GHz. Further investigation through phase angle difference indicated that the antenna structure had three convergence points with a fundamental frequency at 23 GHz, as depicted in Figure 3c. The second and third convergence occurrences were noticed at the second and third harmonic frequencies of 33 and 43 GHz. The phase difference between the modes at these convergence points was close to zero, which was a CA ≈ 180°. It is also worth noting that Modes 1 and 3 and Modes 2 and 4 had similar current characteristics where their maximum equal phase difference of 40° (i.e., 180° ± 40°) was reached between 23 and 33 GHz. This linear phase change from 23 to 33 GHz was expected to generate good impedance matching with a wide bandwidth. A similar impedance matching could be expected from 33 to 43 GHz with a linear maximum equiphase difference of 15°. However, the situation from 33 to 43 GHz was quite different. The surface current *J*_5_ (destructive mode) was lagging by 60° in response to the incident field due to the slow accumulation and discharge of the electric field. This large phase difference impacted the antenna with poor impedance matching from 33 to 43 GHz, so wide bandwidth could not be expected between these frequencies.

**Figure 4:**
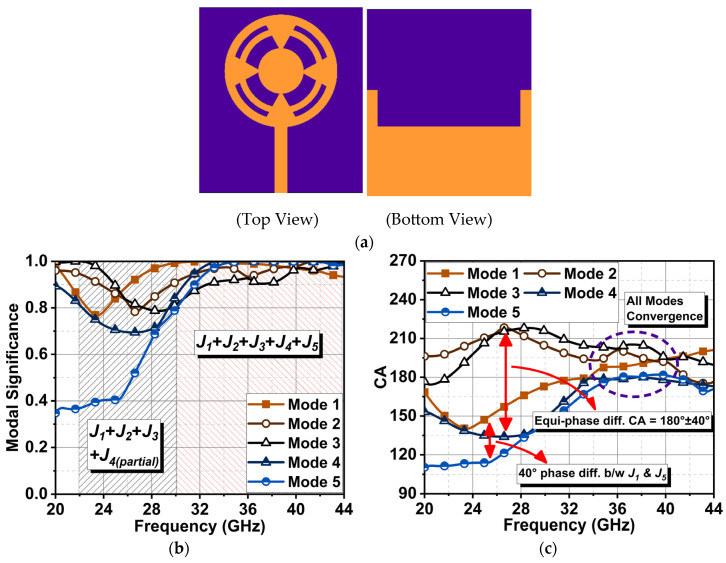
(**a**) Proposed antenna structure (Ant4) with its (**b**) MS and (**c**) CA responses.

**Figure 5:**
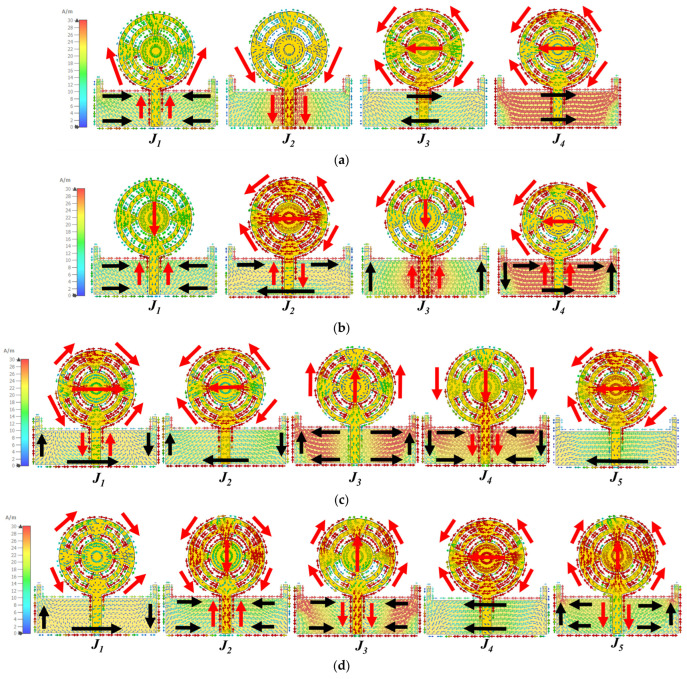
Surface current *J_n_* at (**a**) 25 GHz, (**b**) 28 GHz, (**c**) 35 GHz, and (**d**) 38 GHz.

Further, in the proposed antenna structure, four equilateral triangle-shaped stubs were embedded in the radiator at an angle of 90°, as illustrated in Figure 4a. This modification improved the Mode 5 response from 33 to 43 GHz compared to the earlier antenna structure, as depicted in the MS graph in Figure 4b. In this case, a parallel response of Modes 1 to 4 was noted in response to an incident electric field from 23 to 30 GHz. Similar characteristics continued from 30 to 44 GHz. The characteristic angle showed a linear phase change of modes through Figure 4c. Modes 1 and 4 had an inductance effect, and Modes 2 and 3 had a capacitance effect from 23 to 30 GHz, with a maximum equal-phase difference of 40°. From 30 GHz onwards, all modes converged at a CA ≈ 180°. This response showed that the summation of modes *J*_1_ + *J*_2_ + *J*_3_ + *J*_4*(partial)*_ contributed to resonance from 23 to 30 GHz. From 30 to 44 GHz, the summation of *J*_1_ + *J*_2_ + *J*_3_ + *J*_4_ + *J*_5_ led to resonance. Overall, the structure was estimated to have good impedance matching from 23 to 44 GHz, resulting in a broadband antenna. Furthermore, the radiation patterns were analyzed by considering the surface current distributions of these modes in Figure 5.

The proposed antenna was estimated to generate broadband characteristics with resonance at 28 GHz. The shape of the antenna has a circular ring, so it was expected that the current from the feed line would flow in both directions. The right and left equilateral triangles provide the return path for the current, thereby making the current flow in a clockwise and anti-clockwise direction, generating circular polarization. Therefore, the analysis of CP behavior was also carried out through surface currents of CMA. Due to the antenna’s broadband characteristics, the current behavior was studied at 25, 28, 35, and 38 GHz, as shown in Figure 5. To achieve circular polarization, the surface current of sets of resonant modes should have had equal magnitude with the orthogonal phase. From the MS and CA, it was clear that the sum of *J*_1_ to *J*_4_ contributed to resonance from 23 to 30 GHz. Therefore, the surface current *J*_1_ to *J*_4_ at 25 and 28 GHz is shown in Figure 5a,b. At 25 GHz, *J*_1_ and *J*_2_ had vertical polarization, and *J*_3_ and *J*_4_, horizontal polarization. However, the magnitude of *J*_1_ and *J*_2_ was poor, resulting in a smaller minor axis compared to the major axis of *J*_3_ and *J*_4_. As a result, the polarization was linear on the y-axis. A similar behavior was studied at 28 GHz; *J*_2_ and *J*_4_ had higher magnitudes, leading to linear polarization in the y-axis. However, at 35 and 38 GHz, a pair of modes achieved CP. Looking closely at 35 GHz, *J*_1_ + *J*_2_ + *J*_5_ had y-axis polarization, and *J*_3_ + *J*_4_ had x-axis polarization in Figure 5c. A combination of these orthogonal modes achieved circular polarization. A similar pattern continued at 38 GHz, *J*_1_ + *J*_4_ had y-axis polarization and *J_2_* + *J_3_* + *J_5_* had x-axis polarization, as observed in Figure 5d. 

**Figure 6:**
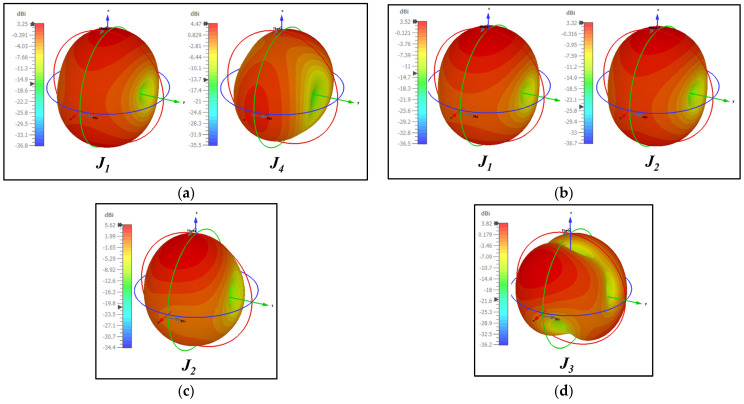
Resultant radiation patterns from surface current *J_n_* at (**a**) 25 GHz, (**b**) 28 GHz, (**c**) 35 GHz, and (**d**) 38 GHz.

Further, an understanding of the resultant radiations from these surface currents is presented in Figure 6. The variations in current distribution with respect to different axes led to different radiation patterns. Here, only the consistent patterns generated by various modes with increasing frequency were considered. The CMA indicated that the proposed structure generated omnidirectional and bidirectional radiation in the XZ and YZ planes, with an approximated gain of 3.5 dBi.

## 3. Antenna Response to Excitation

**Figure 7:**
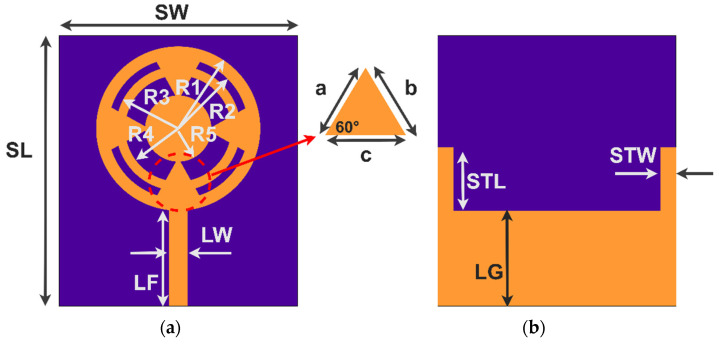
(**a**) Top and (**b**) bottom views of the proposed antenna. Its dimensions in mm are the following: R1 = 1.9, R2 = 1.7, R3 = 1.4, R4 = 1.2, R5 = 0.8, LF = 1.9, LW = 0.45, SL = SW = 6, STL = 0.7, STW = 0.2, LG = 1.7, and a = b = c = 1.1.

**Figure 8:**
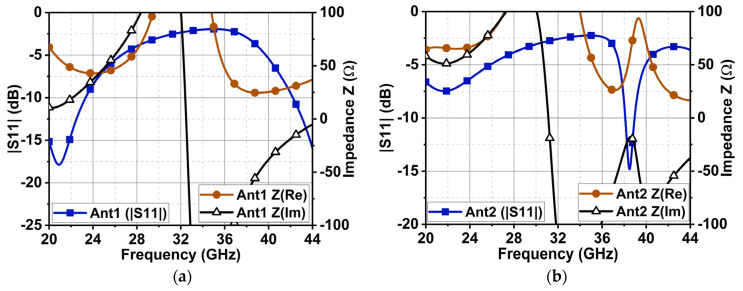

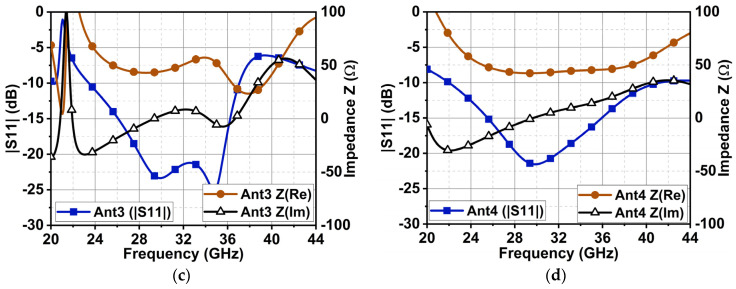
Reflection coefficient and impedance curves of the (**a**) circular ring antenna (Ant1), (**b**) antenna with two circular rings (Ant2), (**c**) antenna with a circular patch at the center and two circular rings (Ant3), and (**d**) proposed antenna (Ant4).

The proposed antenna performance was analyzed with the aid of CMA in Section 2. This section validates the antenna performance in terms of reflection coefficient and impedance matching. Figure 7 shows the dimensions of the antenna structure. Generally, the monopole antenna is half-wavelength or quarter-wavelength; in the case of an antenna on the outer ring with a feed line (Ant1) (Figure 1a), the sum of the circumference of R1 and feed length LF is 13.83 mm which is almost equal to one wavelength. It resulted in resonance at 21.67 GHz, as depicted in Figure 8a. However, the bandwidth is very narrow due to inductive reactance. With another circular ring (R3–R4) added (Ant2) in Figure 2a, a resonance at 38 GHz was observed in Figure 8b, because the circumference length of R3 was one wavelength. Further addition of R5 and four equilateral triangles (Ant4) combined the previous resonances, consequently leading to a broad bandwidth. The impedance was finetuned by varying the ground length, leading to a final bandwidth ranging from 23 to 41 GHz, as shown in Figure 8d. The designed antenna’s overall impedance (Z0) was (50 ± j20) Ω. The structure resulted in a linear change in minor capacitive reactance to inductive reactance. This is due to a combination of capacitive and inductive modes, as presented in Figure 4c.

**Figure 9:**
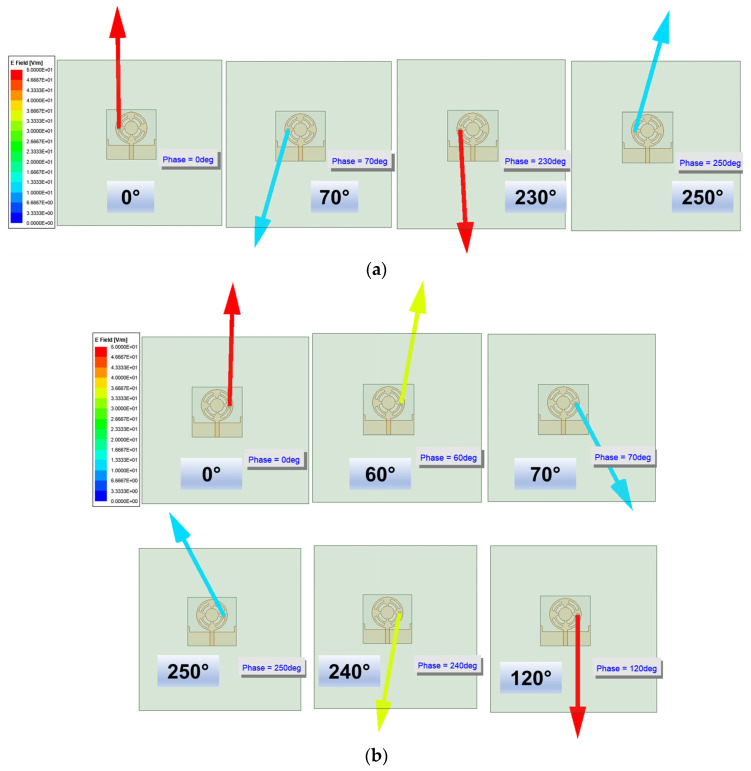
Illustration of circular polarization at 38 GHz. (**a**) LHCP on the left portion of the antenna. (**b**) RHCP on the right portion of the antenna.

The CMA analysis in Section 2 demonstrated that the surface current in the adjacent modes (Figure 5) had an orthogonal phase of 90°, and the summation of these modes resulted in circular polarization. Four equilateral triangles embedded in the design generated this orthogonal phase difference. It perturbed the current flow such that the current in the left portion of the radiator underwent a phase change of +90° and in the right, of –90°. Because of this differential phase change, the left portion of the radiator gave rise to left-handed CP (LHCP) and the right portion, to right-handed CP (RHCP). However, when the resultant tip of an electric field was observed in the far-field region [25], it was realized that the magnitude of the minor axis was lower than the major axis, consequently leading to elliptical polarization. Figure 9 illustrates this behavior of the LHCP and RHCP on the respective left and right portions of the antenna at 38 GHz.

## 4. Simulation and Measurement Results

**Figure 10:**
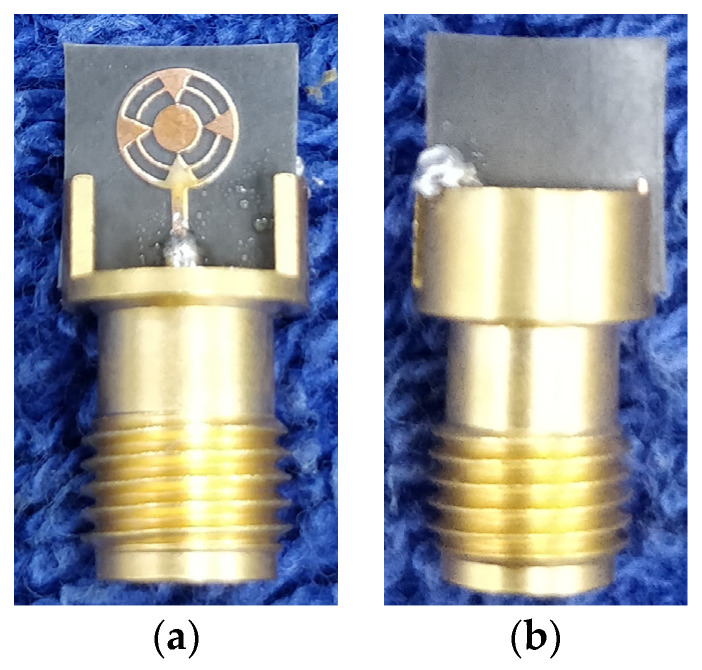
(**a**) Top and (**b**) bottom views of the illustrating prototypical antenna.

**Figure 11:**
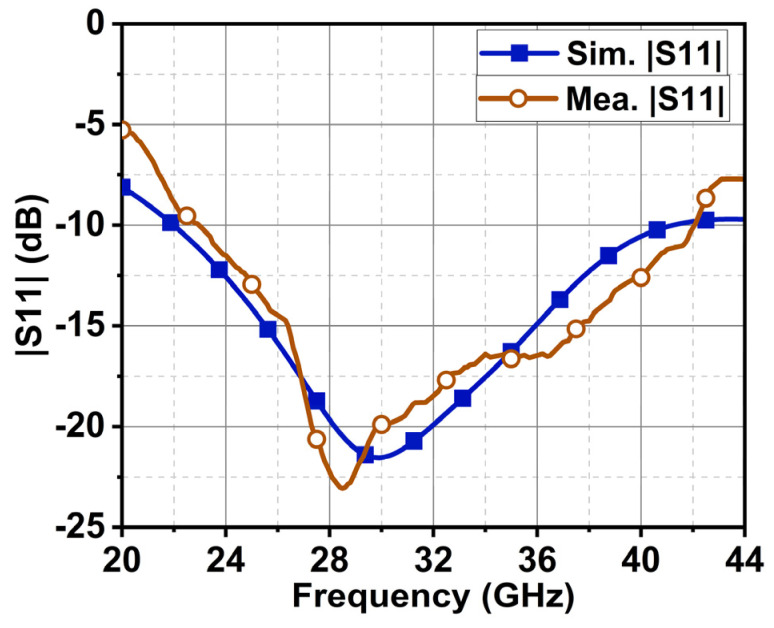
Simulated and measured reflection coefficients of the proposed antenna.

The proposed design was fabricated to validate the simulated results, as shown in Figure 10. A 2.9 mm end launch connector (145-0701-851) was used to feed the signal from a vector network analyzer (Anritsu, Atsugi, Kanagawa, Japan). The VNA was calibrated with short, open, and precision 50 Ω loads before measurement. With this process, the SMA and VNA port impedance would be matched to 50 Ω, causing negligible error in the measured results. The study of antenna structure through CMA indicated that Modes 1 to 4 and Modes 1 to 5 contributed to the resonance from 23 to 30 and 30 to 44 GHz. It was also found that the summation of orthogonal modes from 35 to 38 GHz generated circular polarization. These characteristics were further analyzed through antenna excitation in simulation HFSS (High Frequency Simulation Software) v.21.0 software and from the measurement results. As proof of validation, Figure 11 illustrates the simulated and measured reflection coefficient |S11|. The achieved simulation and measurement bandwidths were 19.2 GHz (22–41.2 GHz) and 19 GHz (23–42 GHz), as depicted in Figure 11. 

**Figure 12:**
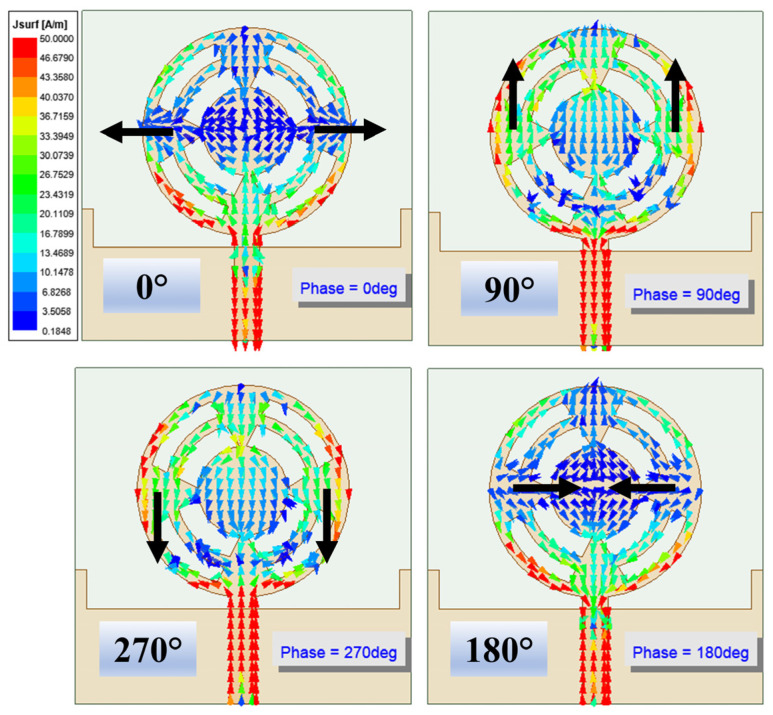
Surface current *J_n_* for port excitation. The flow current depicts the RHCP and LHCP characteristics on the left and right portions of the antenna, respectively.

The circular antenna structure splits the input current in both directions. Further, the four equilateral triangles, which connect the outer ring to the center circular patch, provide the return path to the current. Thus, the current flowing in the left and right of the radiator undergoes a ±90° phase shift, resulting in RHCP and LHCP. These behaviors were studied in Section 3 with the E-field locus in the far-field region. This same behavior is also illustrated through the surface current in Figure 12 at 38 GHz. In the left portion of the antenna, the surface current changed its direction clockwise, and the left portion of the antenna was counterclockwise. The direction of the current on the conductor and the radiated E-field have opposite polarities, which can be understood from Equation (5):

(5) $$E=\int_{Surface}\frac{-j\omega \mu e^{-jkR}}{4\pi R}J_{s}ds\;$$

where ω is the angular frequency, μ is free space permittivity, *k* is the wavenumber, and *R* is the far-field distance. 

**Figure 13:**
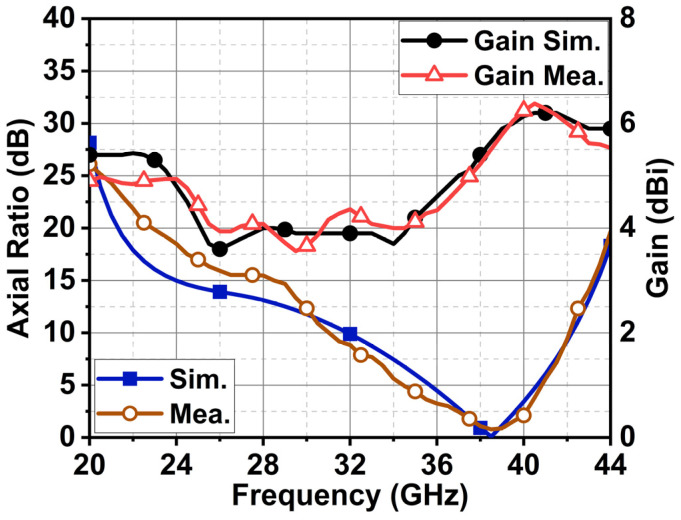
Simulated and measured axial ratio and maximum gain.

**Figure 14:**
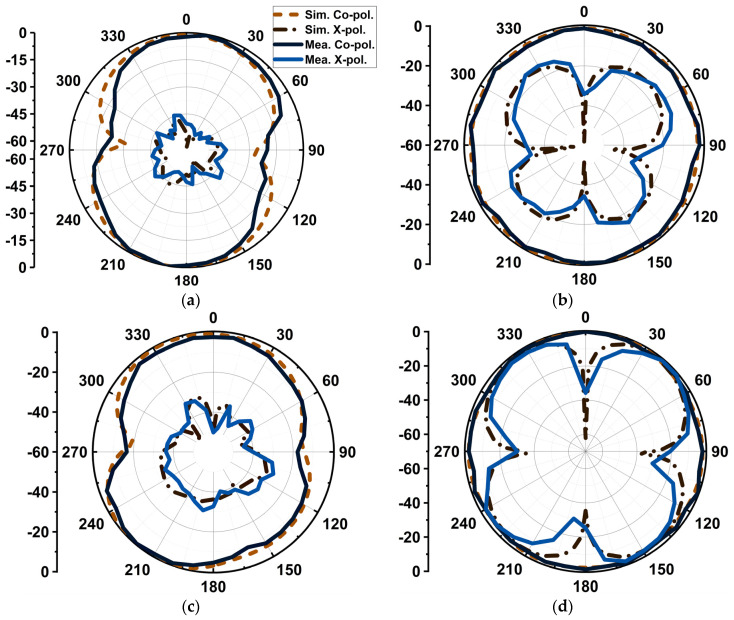
Simulated and measured radiation patterns. (**a**) XZ plane at 28 GHz, (**b**) YZ plane at 28 GHz, (**c**) XZ plane at 38 GHz, and (**d**) YZ plane at 38 GHz.

The antenna’s −3 dB axial ratio bandwidth was 3.5 GHz (36.5–40 GHz) from the simulation. The measured axial ratio bandwidth was 3.7 GHz (36.5–40.2 GHz), as displayed in Figure 13. The antenna resulted in a maximum gain of 6.2 dBi with an average gain of 4.75 dBi throughout its bandwidth. The proposed design has partial ground, due to which its radiation resulted in omnidirectional XZ and YZ planes, as displayed in Figure 14. Cross-polarization was minimal in the order of −45 dB in the XZ plane. However, in the YZ plane, the cross-polarization was −17 dB and −2 dB due to the circular polarization characteristics of the antenna at 28 and 38 GHz, respectively.

## 5. Link Budget Estimation for IoT Indoor Scenario

**Figure 15:**
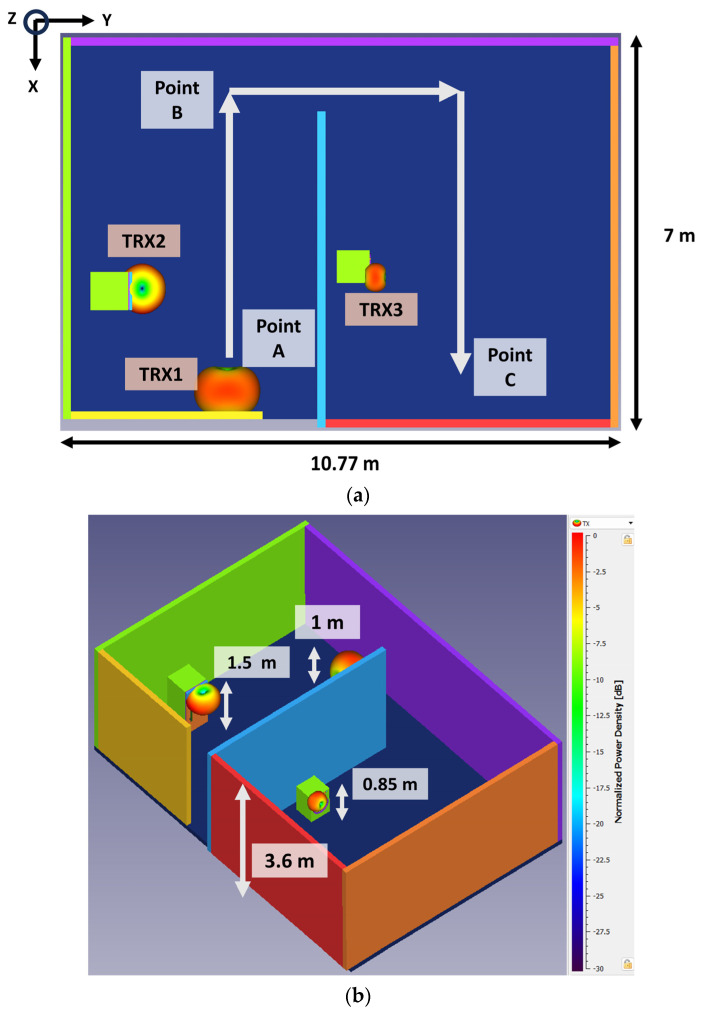
(**a**) 2D and (**b**) 3D simulation environment representation for link margin estimation.

The proposed antenna achieved omnidirectional radiation with CP and an average gain of 4.75 dBi. From this achieved gain and radiation pattern, the designed antenna is suitable for IoT applications in indoor scenarios. The transceiver performance of the designed antenna between different IoT devices such as mobile, fridge, and washing machines is studied in this section. The propagation of millimeter-wave signals experiences drastic attenuation for the first meter of distance [26]. Therefore, it becomes essential to estimate the losses for indoor scenarios. In our analysis, a carpet area of 75.39 m^2^ was considered. The area was partitioned into two rooms connected by a corridor path, as demonstrated in Figure 15. In calculating the link margin, the reflection from the wall of a 3.6 m height, reflection from objects, and path loss exponents were considered. For simplicity, the rooftop effect was neglected in the analysis. The TRX1, TRX2, and TRX3 were a mobile phone, fridge, and washing machine. The TRX2 and TRX3 were assumed to be stationary, whereas TRX1 moved from point A to B, B to C, and then to D (relative distance (Rel. Dis.) was considered instead of the actual distance between devices), as depicted in Figure 15a. The TRX1, TRX2, and TRX3 antennas were assumed to be in the XY, YZ, and XY planes, respectively. With this setup, the transmission and reception power between the antennas were well studied for circular polarization behavior. The TRX1, TRX2, and TRX3 were at heights of 1 m, 1.5 m, and 0.85 m, respectively, as shown in Figure 15b. The analysis was carried out for two cases: (i) the communication between TRX1 and TRX2, where the antennas were in a cross-polarization setup and (ii) the communication between TRX1 and TRX 3, where the antennas were in co-polarization. 

The simulation was performed using the HFSS savant tool. The estimated received power at TRX2 and TRX3 was calculated using Equation (6) [27]:

(6) $$P_{TRXn}\left(\mathrm{dB}\right)=P_{TRX1}\left(\mathrm{dB}\right)+G_{TRX1}\left(\mathrm{dB}\right)+G_{TRXn}\left(\mathrm{dB}\right)-P_{Loss}\;\left(\mathrm{dB}\right)$$

where, *n* = 2 and 3, and $G_{TRX1}$ and $G_{TRXn}$ are the antenna gains of TRX1, 2, and 3, which were considered based on the measurement results of the antenna. $P_{Loss}$ is the path loss at 28 GHz. The effects of the line-of-sight (LOS) and non-line-of-sight (NLOS) conditions were also studied in estimating the link margin. When TRX1 moved from point A to B and then to mid-B to C, it had an LOS condition with TRX2; beyond this point, they both underwent an NLOS scenario. The same applied to TRX1 and TRX3; they had an LOS from point D to C until mid-C to D and later entered into the NLOS condition. Considering an ideal binary phase-shift keying (BPSK) modulation, the actual required power ($RP_{TRXn})$ at TRX2 and TRX3 was calculated using Equation (7) [27]:

(7) $$RP_{TRXn}=\frac{E_{b}}{N_{0}}+KT+B_{r}\;\left(\mathrm{dB}\right)$$

where $\frac{E_{b}}{N_{0}}$ is the symbol-to-noise ratio in the case of BPSK, *K* is Boltzmann’s constant, *T* is the temperature in Kelvin, and *B_r_* is the varied increased bit rate. 

**Figure 16:**
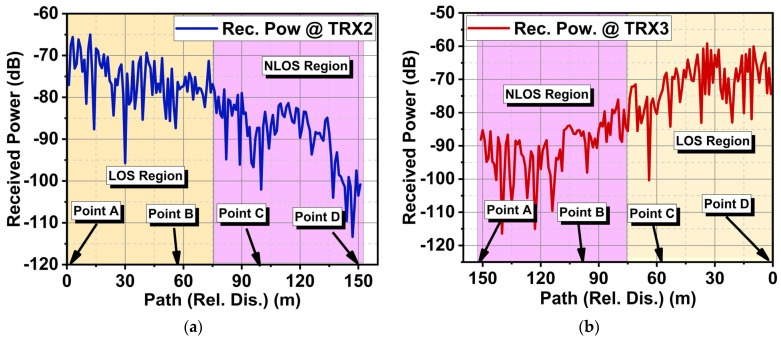
Computed received signals for (**a**) Case I and (**b**) Case II.

**Figure 17:**
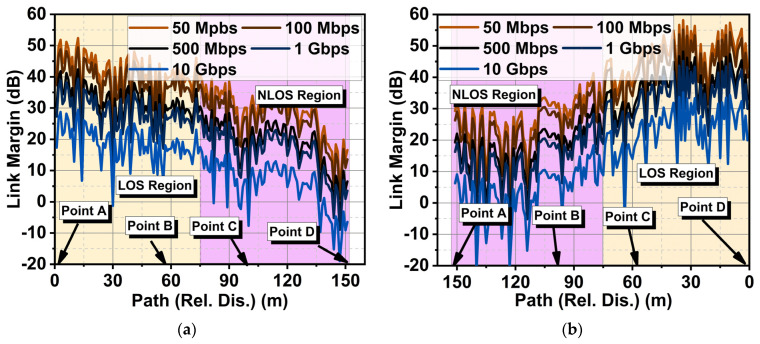
Link margin estimation of the proposed antenna for IoT indoor scenarios for (**a**) Case I and (**b**) Case II.

Figure 16a,b present the computed received signal between TRX1 and TRX2 (Case I) and between TRX1 and TRX3 (Case II). The received signal strength for Case II was higher than for Case I due to co-polarization. In Case I, the received signal strength was relatively weak due to cross-polarization. However, the proposed CP antenna did better even in this scenario. The link margin estimation results for Case I in Figure 17 indicated that TRX1 and TRX2 had good LOS conditions from point A to B until mid-B to C, after which a significant drop in received power was observed. Consequently, a drop in the link margin also took place. A sudden surge and drop in the graph were due to the constructive and destructive effects of multipath signals. TRX1 and TRX3 had a good LOS from Point D to C until mid-C to B. From this point onwards, they experienced NLOS conditions. In both cases, the antenna could deliver 10 Gbps of data under LOS conditions until a 75 m distance if the link margin was 10 dB. This study indicated that due to the circular polarization of the antenna, the variation in link margin estimation for LOS in both cases was negligibly minimal. Under the NLOS scenario, Case II performed relatively well compared to Case I. This is due to the lower magnitude of the minor axis in CP, which gave rise to elliptical polarization. This means that in NLOS scenarios, the antenna with elliptical polarization had a slight impact on the performance. 

## 6. Comparative Analysis

As discussed in the introduction, a broadband antenna with CP can be achieved with ME, SIW, and DRA antennas. However, to achieve the same with the planar patch antenna is quite challenging. This article proposes a broadband antenna operating from 23 to 42 GHz with a −3 dB axial ratio bandwidth of 4.2 GHz (36–40.2 GHz), which is comparable with the existing state-of-the-art designs in Table 1. Also, the proposed design is low-profile compared to others in the table. The antenna represents RHCP and LHCP behavior, with a decent gain of 4 to 6.2 dBi. The developed antenna was validated for IoT indoor conditions, for which other designs have not been performed. 

## 7. Conclusions

This article presents a low-profile broadband antenna covering the Ka-band for millimeter-wave IoT applications. Its circular ring-shaped structure with equilateral triangles provides a path for surface current to complete its loop with the differential phase, thereby generating circular polarization. The CMA analysis showed how the summation of orthogonal modes leads to CP. The antenna was verified for IoT application in indoor scenarios, and the results showed a satisfactory link margin of 10 dB to a distance of 75 m with a data rate of 10 Gbps. Further, the gain of the antenna can be enhanced with array techniques. Also, its data rate can be improved with MIMO antennas. Therefore, the proposed antenna is suitable for mmWave applications.

## Figures and Tables

**Table 1:** Performance comparison with other designs.

Ref.	Dim. in λ	Dim. in mm^2^	Ant Type	Res. in GHz	BW in GHz	AR in GHz	Gain	Pol.	LM
[13]	0.62 × 0.45	6.8 × 5	Parasitic patch	27	21–28.5	–	4	LP	No
[14]	1.1 × 1.1	12 × 12	Patch + Metasurface	27	24.25–29.5	25.9–28.2	7	RHCP/LHCP	No
[28]	1.03 × 1.03	11 × 11	Metasurface	28	26.2–30.8	–	6.5–10.1	LP	No
[29]	0.65 × 0.7	3.25 × 3.5	Patch	60	55–64	–	4	LP	No
[30]	0.8 × 0.8	4 × 4	Patch	60	58–90	–	5–6.7	LP	No
[31]	0.47 × 0.28	5 × 3	Patch	28	26–30	–	3.7–4.7	LP	No
[32]	1.12 × 1.12	12 × 12	Monopole	28	26–29	–	4.8	LP	No
[33]	2.1 × 1.4	22.5 × 15	Patch dipole	28	25.6–29.5	–	1	LP	No
[34]	2.33 × 2.33	25 × 25	Period stub-loaded	28	27.5–32.5	25.3–32.7	NA	CP	No
Prop.	0.56 × 0.56	6 × 6	Patch	28	23–42	36–40.2	4–6.2	RHCP/LHCP	Yes

Note: Dim.—dimensions, Ant—antenna, Res.—resonance, BW—bandwidth, AR—axial ratio, LM—link margin.

## Data Availability

Data are contained within the article.
